# High Risk First Degree Relatives of Type 1 Diabetics: An Association with Increases in CXCR3^+^ T Memory Cells Reflecting an Enhanced Activity of Th1 Autoimmune Response

**DOI:** 10.1155/2014/589360

**Published:** 2014-03-23

**Authors:** Tanja Milicic, Aleksandra Jotic, Ivanka Markovic, Katarina Lalic, Veljko Jeremic, Ljiljana Lukic, Natasa Rajkovic, Dušan Popadic, Marija Macesic, Jelena P. Seferovic, Sandra Aleksic, Jelena Stanarcic, Milorad Civcic, Nebojsa M. Lalic

**Affiliations:** ^1^Clinic for Endocrinology, Diabetes and Metabolic Diseases, Clinical Centre of Serbia, Faculty of Medicine, University of Belgrade, Dr Subotica 13, 11000 Belgrade, Serbia; ^2^Institute for Biochemistry, Faculty of Medicine, University of Belgrade, Dr Subotica 8, 11000 Belgrade, Serbia; ^3^Department for Operations Research and Statistics, Faculty of Organizational Sciences, University of Belgrade, Jove Ilica 154, Belgrade, Serbia; ^4^Institute of Microbiology and Immunology, Faculty of Medicine, University of Belgrade, Dr Subotica 8, 11000 Belgrade, Serbia

## Abstract

We analyzed the level of (a) CXCR3^+^ (Th1) and CCR4^+^ (Th2) T memory cells (b) interferon-**γ** inducible chemokine (IP-10)(Th1) and thymus and activation-regulated chemokine (TARC)(Th2), in 51 first degree relatives (FDRs) of type 1 diabetics (T1D) (17 high risk FDRs (GADA^+^, IA-2^+^) and 34 low risk FDRs (GADA^−^, IA-2^−^)), 24 recent-onset T1D (R-T1D), and 18 healthy subjects. T memory subsets were analyzed by using four-color immunofluorescence staining and flowcytometry. IP-10 and TARC were determined by ELISA. High risk FDRs showed higher levels of CXCR3^+^ and lower level of CCR4^+^ T memory cells compared to low risk FDRs (64.98 ± 5.19 versus 42.13 ± 11.11; 29.46 ± 2.83 versus 41.90 ± 8.58%, resp., *P* < 0.001). Simultaneously, both IP-10 and TARC levels were increased in high risk versus low risk FDRs (160.12 ± 73.40 versus 105.39 ± 71.30; 438.83 ± 120.62 versus 312.04 ± 151.14 pg/mL, *P* < 0.05). Binary logistic regression analysis identified the level of CXCR3^+^ T memory cells as predictors for high risk FDRs, together with high levels of IP-10. The results imply that, in FDRs, the risk for T1D might be strongly influenced by enhanced activity of Th1 and diminished activity of Th2 autoimmune response.

## 1. Introduction

It has been shown that first degree relatives (FDRs) of patients with type 1 diabetes (T1D) have at least 10 times higher relative risk of T1D than in the general population [1, 2]. Until now, detection of *β*-cell antibodies represents relevant clinical tool for prediction of T1D and established risk factor for T1D development among FDRs [3–6].

However, it is still difficult to define impairments in cellular immunity and determine the potential cellular risk markers for progression in T1D in FDRs. Previously it was shown that changes in functionally different memory Th1/Th2 subsets might be involved in the pathogenesis of T1D [7] and that those changes might be accompanied by the expression of different chemokine receptors [7, 8]. In that context, CXC chemokine receptor 3 (CXCR3) is selectively expressed on memory Th1 cells, while CC chemokine receptor 4 (CCR4) is expressed on memory Th2 cells [8, 9]. Simultaneously, it was demonstrated that one of ligands for CXCR3 receptor is chemokine interferon *γ* inducible protein (IP-10) [9–12], while CCR4 receptor attracts the thymus and activation-regulated chemokine (TARC) [11]. Also, it was shown that chemokine receptors, together with their ligands, might be involved in different Th1/Th2 cell migratory capacity and the extravasation of T cells into inflamed tissue [13, 14].

Recently, the studies of the role of CXCR3^+^ and CCR4^+^ T cells in the pathogenesis of T1D have suggested the importance of pancreatic *β* cells infiltration with CXCR3^+^ T cells in T1D patients [15–19], while the role of CCR4^+^ T cells in T1D still remains controversial [20]. The infiltration with CXCR3^+^ T cells was found to be simultaneous to the reduced expression of Th1 associated chemokine receptors on T cells in the peripheral blood, prior to the onset of T1D [21].

Having that in mind, we examined the level of CD4^+^ T cells subsets expressing those Th1 or Th2-associated chemokine receptors and circulating chemokines in FDRs, in order to characterise cellular immunity profile of FDRs and evaluate their possible role in the T1D developing.

Therefore, the aim of this study was to analyze the percentage of the (a) CXCR3^+^ (Th1 associated) and CCR4^+^ (Th2 associated) subsets of T memory cells and (b) the level of chemokines IP-10 (Th1 type) and TARC (Th2 type), in nondiabetic FDRs, previously defined in subgroups of high versus low risk FDRs, recent-onset T1D patients (R-T1D), and healthy controls.

## 2. Subjects and Methods

### 2.1. Patients/Research Design

We examined 51 FDRs of the patients with T1D, 24 patients with R-T1D, and 18 healthy unrelated control subjects. FDRs of patients with T1D (29 females and 22 males, median age: 27,57 ± 7,21 years) were siblings and/or parents, aged up to 45 years. We divided them into 2 subgroups according to presence/absence of autoantibodies; 17 of them was classified as high risk (hr) FDRs, who were persistently positive for the presence of glutamic acid decarboxylase (GADA) and tyrosine phosphatase insulinoma antigen-2 (IA-2A), while 34 FDRs were classified as low risk (lr) FDRs, negative for both autoantibodies.

All participants did not show signs of other acute and chronic diseases which could influence glucose homeostasis and had no infective, allergic, and autoimmune diseases during 6 months before blood samples were taken. Moreover, all the participants in this study did not use immunomodulatory drugs at least 3 months before investigation.

All 24 patients with R-T1D fulfilled the diagnostic criteria presented with persistent hyperglycemia, proneness to develop ketoacidosis, polyuria, polydipsia, weight loss, and symptoms consistent with underlying deficiency in insulin secretion, according to the criteria set out by the Expert Committee of American Diabetes Association [22]. The diagnosis was confirmed by the presence of autoantibodies to GADA, and/or IA-2A. Blood samples were collected within 3 months of diagnosis and initiation of intensified insulin therapy after patients were admitted at Clinic for Endocrinology, Diabetes and Metabolic Diseases. We tested patients at the onset of T1D in insulin requiring state (IRS).

IRS in patients with R-T1D was defined as necessity for insulin therapy in order to accomplish euglycemia (fasting glycemia <5,5 mmol/L, postprandial glycemia <10.0 mmol/L). All the patients were treated with intensified insulin therapy, multiple daily (subcutaneous) injection, 4 daily doses, human rapid acting insulin (Actrapid HM 100, Novo Nordisk) before the meals and in the evening at bedtime, and NPH insulin (Insulatard HM 100, Novo Nordisk).

Inclusion criteria for 18 control subjects were blood glucose less than 110 mg/dL (normal levels), no history of T1D in the family, and absence of T1D-specific antibodies.

Only nondiabetic FDRs and controls were included, which was verified by using a 2-hour 75 g oral glucose tolerance test (OGTT) [23].

According to the study design, all FDRs were tested for the presence of GADA and IA-2A, twice during one year.

The investigation and detection of autoantibodies in FDRs and R-T1D were performed in the Clinic for Endocrinology, Diabetes and Metabolic Diseases, after patients gave the informed consent to participate in the study. Isolation of peripheral blood mononuclear cells and detection of surface markers of cellular immunity and chemokines levels were conducted at the Institute for Biochemistry Faculty of Medicine University of Belgrade. The study was approved by the Institutional Review Board.

### 2.2. Detection of GADA and IA-2

GADA and IA2 were measured using radioimmunoassay method, according to the manufacturer's instructions (CIS Bio international, Gif Sur Yvette, France). The assay was performed in duplicate for the standards, controls, and samples. The interassay coefficients of variation were 4.9%, 7% and 3.3, 5.3%, for the GADA and IA2, respectively, and intra-assay coefficients of variation were 3.6%, 3.7% and 6.4 and 15.1, for all assays, respectively. Values higher than 1 U/mL were considered as positive.

### 2.3. Immunofluorescence Staining and Flow Cytometry Analysis

Patients' peripheral blood samples were obtained by venipuncture into heparinized vacutainers (BD). 100 microL of whole blood was incubated with saturating quantities of the appropriate FITC-, PE-, PerCP-, and APC-conjugated monoclonal antibodies for 30′ in the dark at room temperature. Isotype-matched FITC, PE-, PerCP- and APC-conjugated irrelevant monoclonal antibodies were used as negative controls. After staining, erythrocyte lysis was performed using FACS Lysing solution (BD) and the cells were subsequently washed twice in PBS and then fixed with 1% paraformaldehyde solution in PBS.

Labeled cells were analyzed in a FACSCalibur flow cytometer (BD Biosciences, San Diego, CA, USA) equipped with an air-cooled argon ion laser that operated at 488 nm and a red diode laser that operated at 632 nm. The cells were collected (1–3 × 10^4^ events acquired per test) and analyzed using the CellQuestPro software (BD Biosciences), and the percentage and mean fluorescence intensity of the cells for each examined molecule were monitored. Lymphocytes were gated by their forward- and side-scatter properties. Chemokine receptor expression profile was determined for the CD4^+^CD45RO^+^ cells (using the FL3-FL4 dot plot) belonging to the lymphocyte gate. The color combination commonly used in the present study was PercP-anti-CD4 (BD Pharmingen, San Diego, CA, USA), APC-anti-CD45RO (BD Pharmingen, clone UCHL1, San Diego, CA, USA), FITC-anti CXCR3 (R&D Systems, clone 49801, Minneapolis, MN, USA), and PE-anti CCR4 (BD Pharmingen, clone 1G1, San Diego, CA, USA).

### 2.4. Chemokine Analysis

Serum levels of human IP-10 and TARC were determined using quantitative sandwich enzyme immunoassays (R&D Systems, Minneapolis, MN, USA). All the analyses were performed in duplicate. The assay was performed according to the manufacturer's instruction.

### 2.5. Statistics

Data are presented as mean ± SD. Data were tested for normal distribution using Kolmogorov-Smirnov test. The chemokine receptor and chemokine levels were compared with ANOVA with Bonferonni/Tamhane correction analysis. Binary logistical regression analysis was performed. Correlation was estimated by Pearson's (*r*) correlation coefficient. Two-tailed *P* values less than 0.05 were considered significant. Data were analyzed using the Statistical Package for the Social Sciences (SPSS) software (Advanced Statistics, version 17.0), Chicago, IL.

## 3. Results

### 3.1. Clinical Characteristics

Summary of baseline clinical characteristics of all participants included in the study is shown in Table 1.

### 3.2. Flow Cytometric Detection of CD4^+^ Memory T Cell Subsets

It has been suggested that human macrophages and B cells can express CD4, therefore we gated on CD3^+^ cells, which is associated with T cells. In different panels, memory T cells are identified as CD45RO^+^ (8). Quadrants were set according to the staining of control mAbs. Gated peripheral blood human T cells coexpressing CD4 and CD45RO were stained for cell surface expression of CXCR3 and CCR4. Having in mind the conflicting role of CD4lo T cells in T1D, we have additionally analyzed CD4hi/CD4lo panel in our groups of subjects, showing low level of CD4lo subpopulation in lymphocytic gate (hrFDRs: 1.03 ± 0.73%, lrFDRs 1.23 ± 1.06%, RT1D: 0.96 ± 0.57%, HC 1.25 ± 1.52%, *P* = NS) and among CD4^+^ T cells (hrFDRs 2.67 ± 0.55%, lrFDRs 3.17 ± 2.66%, RT1D 2.24 ± 1.37%, HC 3.05 ± 3.89%, *P* = NS), range (0–6.77% and 0–17.32%, resp.). In that context, our further analysis was based only on CD4hi, that is, CD4^+^CD45RO^+^ T cells, in order to avoid the overestimation of the effect of CD4lo subpopulation on final results.

CD4^+^ memory T cells were quantified by flow cytometry in hr and lr nondiabetic FDRs, patients with R-T1D in IRS, and healthy controls, in order to examine if receptor expression was different at various stages of development of T1D. Hr FDRs were positive for the presence of GADA and IA-2A, while lr FDRs were negative for both autoantibodies.

Analyses of the percentages of T memory (CD4^+^CD45RO^+^) cells in hr and lr nondiabetic FDRs, patients with sa R-T1D, and healthy controls revealed no differences (CD4^+^CD45RO^+^: 28.18 ± 5.63 versus 25.03 ± 6.26 versus 27.23 ± 8.54 versus 27.67 ± 6.59%, resp., *P* = NS).

**Figure 1:**
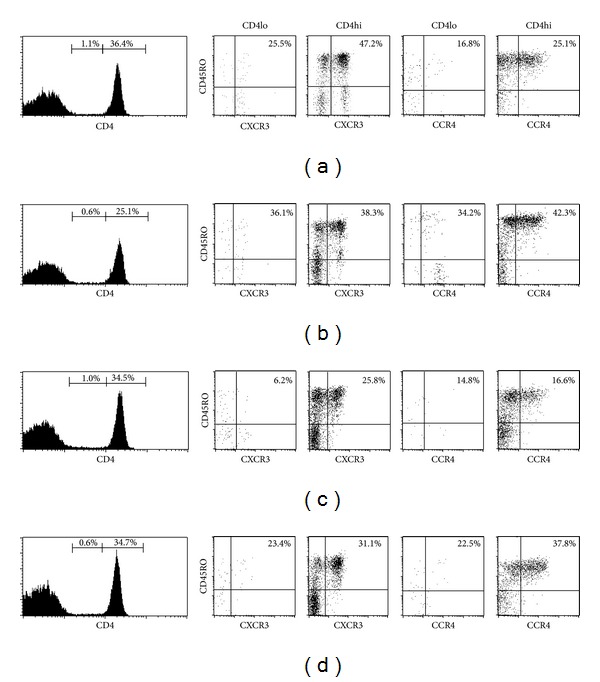
Expression profiles of CXCR3 and CCR4 on CD4 T cells subpopulations (CD4^+^ low and CD4^+^ high). Based on their expression profile, CD4^+^ T cells were divided into CD4^+^ low and CD4^+^ high subpopulations, and the percentage of their contribution to the lymphocyte gate was displayed ((a)–(d), far left panel) and CXCR3 and CCR4 expression was determined for CD4^+^ lo/CD45RO^+^ cells and CD4^+^ high/CD45RO^+^ cells. Representative histograms and dot plots for (a) high risk first degree relatives (hrFDR), (b) low risk first degree relatives (lrFDR), (c) patients with recent-onset type 1 diabetes in insulin requiring state (RT1D-IRS), or (d) healthy control (HC) are presented.

**Figure 2:**
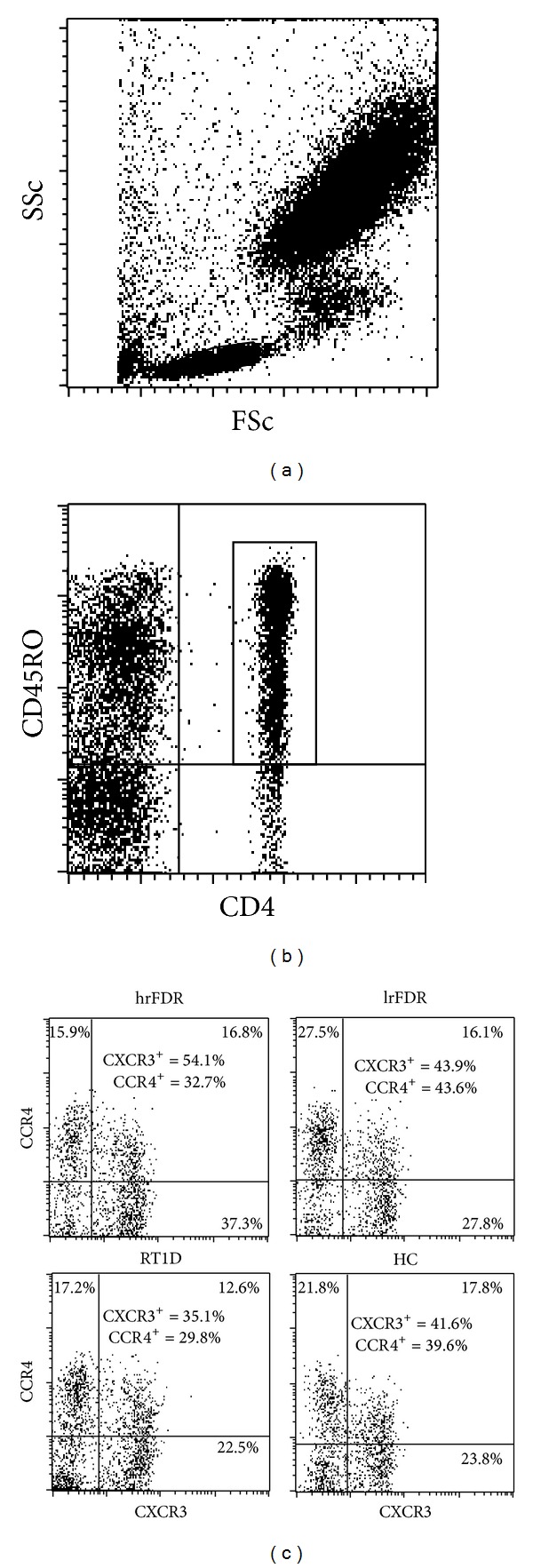
Expression profiles of CXCR3 and CCR4 on circulating CD45RO^+^ and CD4^+^ T cells. (a) Cells from lymphocyte gate were selected and (b) population of CD4^+^/CD45RO^+^ cells was chosen for chemokine receptor analysis. (c) Representative dot plots of CXCR3 and CCR4 expression on CD4^+^/CD45RO^+^ cells from analysis of high risk first degree relatives (hrFDR), low risk first degree relatives (lrFDR), patients with recent-onset type 1 diabetes in insulin requiring state (RT1D-IRS), or healthy control (HC) blood sample following the 4-color staining using corresponding mAbs and flow-cytometric analysis. The results show the percentage of CD45RO^+^/CD4^+^ cells expressing CXCR3 and CCR4 in each representative sample.

Representative histograms and dot plots for each group analyzed for expression profiles of CXCR3 and CCR4 on CD4 T cells subpopulations (CD4^+^ low and CD4^+^ high) are presented (Figure 1), as well as representative dot plots of CXCR3 and CCR4 receptors expression on CD4^+^ CD45RO^+^ T cells (Figure 2).

### 3.3. Prevalence of CXCR3^+^ T Memory Cells in Peripheral Blood

**Figure 3:**
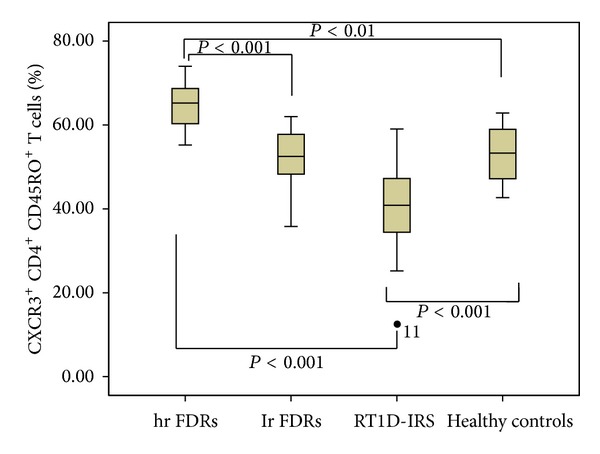
The level of CXCR3^+^CD4^+^CD45RO^+^ T cells in the peripheral blood of first degree relatives (FDRs) at high risk (hr FDRs) and low risk (lr FDRs) for development of type 1 diabetes, patients with recent-onset type 1 diabetes in insulin requiring state (RT1D- IRS), and healthy controls. Expression of CXC chemokine receptor 3 (CXCR3) chemokine receptors on CD4^+^ cells was determined by flow cytometry and shown in box-plots. Horizontal lines represent the median; the box comprises the 25th and 75th percentiles and the error bars comprise the 10th and 90th percentiles. Outliers are indicated. Receptor levels are expressed as percentage chemokine receptor-positive cells of CD4^+^CD45RO^+^ T cells. Comparison among hr FDRs, lr FDRs, patients with RT1D-IRS, and healthy controls (ANOVA test).

We found that percentage of CD4^+^CD45RO^+^ cells expressing CXCR3, Th1-associated receptor, was significantly higher in hr FDRs compared to lr FDRs, patients with R-T1D in IRS, and healthy controls (64.98 ± 5.19 versus 42.13 ± 11.11 versus 40.19 ± 11.52 versus 53.09 ± 6.29%; resp., hr FDRs versus lr FDRs: *P* < 0.001; hr FDRs versus R-T1D IRS: *P* < 0.001; hr FDRs versus healthy controls: *P* < 0.01) (Figure 3).

On the other hand, the percentage of CXCR3^+^ T memory cells was significantly lower in patients with R-T1D in comparison to healthy controls (*P* < 0.001) (Figure 3).

### 3.4. Prevalence of CCR4^+^ T Memory Cells in Peripheral Blood

**Figure 4:**
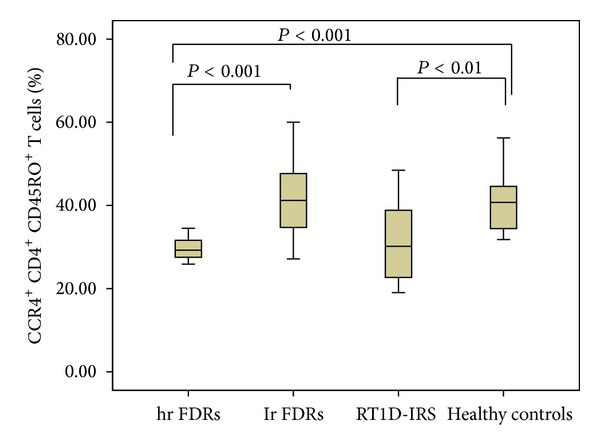
The level of CCR4^+^CD4^+^CD45RO^+^ T cells in the peripheral blood of first degree relatives (FDRs) at high risk (hr FDRs) and low risk (lr FDRs) for development of type 1 diabetes, patients with recent-onset type 1 diabetes in insulin requiring state (RT1D-IRS), and healthy controls. Expression of CC chemokine receptor 4 (CCR4) chemokine receptors on CD4^+^ cells was determined by flow cytometry and shown in box-plots. Horizontal lines represent the median; the box comprises the 25th and 75th percentiles and the error bars comprise the 10th and 90th percentiles. Outliers are indicated. Receptor levels are expressed as percentage chemokine receptor-positive cells of CD4^+^CD45RO^+^ T cells. Comparison among hr FDRs, lr FDRs, patients with RT1D-IRS, and healthy controls (ANOVA test).

We found that percentage of CD4^+^CD45RO^+^ T cells expressing CCR4, Th2-associated receptor, was significantly lower in hr FDRs compared to lr FDRs and healthy controls (29.46 ± 2.83 versus 41.90 ± 8.58 versus 40.90 ± 7.24%; resp., *P* < 0.001) (Figure 4). Simultaneously, the percentage of those cells was similar in hr FDRs and patients with R-T1D in IRS (29.46 ± 2.83 versus 31.53 ± 9.67%, *P* = NS) (Figure 4).

In addition, the percentage of CCR4^+^ T memory cells was significantly lower in patients with R-T1D in IRS in comparison to healthy controls (*P* < 0.01) (Figure 4).

We did not find correlation between the level of GAD and IA-2 antibodies and percentage of CXCR3^+^ and CCR4^+^ T memory cells, neither in hr FDRs nor in lr FDRs (data not shown).

### 3.5. Chemokine Levels in the Peripheral Blood

It has been reported that the levels of circulating chemokines are markers of cellular immunity. Therefore, we analyzed the levels of IP-10, Th1-associated, and TARC, Th2-associated chemokines, in hr and lr FDRs, in patients with R-T1D in IRS, and in healthy controls.

**Figure 5:**
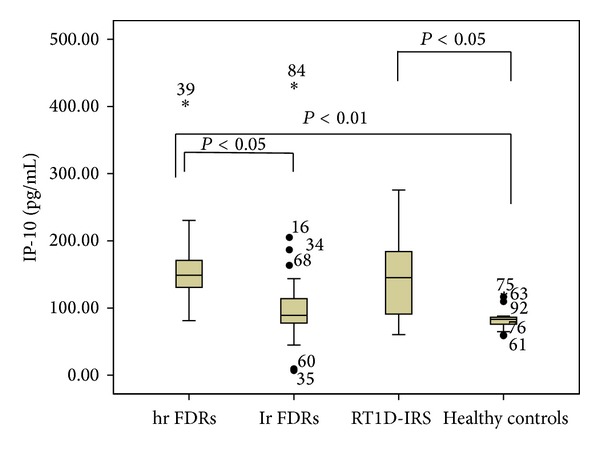
The level of IP-10 in the peripheral blood of first degree relatives (FDRs) at high risk (hr FDRs) and low risk (lr FDRs) for development of type 1 diabetes, patients with recent-onset type 1 diabetes in insulin requiring state (RT1D- IRS), and healthy controls. The level of IP-10 was determined by ELISA and shown in box-plots. Horizontal lines represent the median; the box comprises the 25th and 75th percentiles and the error bars comprise the 10th and 90th percentiles. Outliers are indicated. Comparison among hr FDRs, lr FDRs, patients with RT1D-IRS, and healthy controls (ANOVA test).

The analysis of the circulating IP-10 level has shown that the level of IP-10 in hr FDRs was significantly higher compared with lr FDRs and healthy controls, and similar to the level detected in patients with R-T1D in IRS (160.12 ± 73.40 versus 105.39 ± 71.30 pg/mL *P* < 0.05; 160.12 ± 73.40 versus 85.24 ± 19.82 pg/mL *P* < 0.01; 160.12 ± 73.40 versus 141.99 ± 69.59 pg/mL, *P* = NS) (Figure 5). In addition, the level of IP-10 was significantly higher in patients with R-T1D in IRS in comparison to healthy controls (*P* < 0.05) (Figure 5).

**Figure 6:**
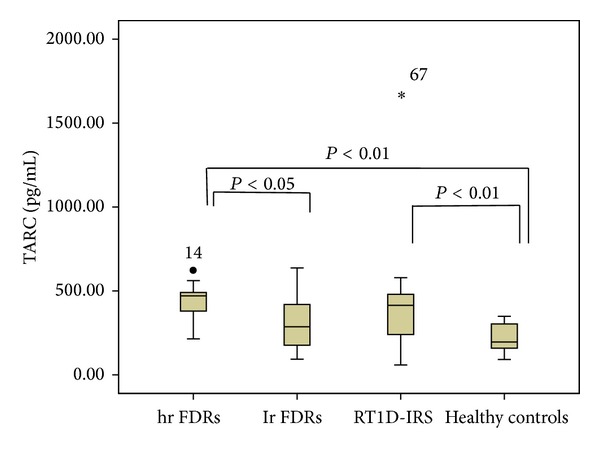
The level of TARC in the peripheral blood of first degree relatives (FDRs) at high risk (hr FDRs) and low risk (lr FDRs) for development of type 1 diabetes, patients with recent-onset type 1 diabetes in insulin requiring state (RT1D-IRS), and healthy controls. The level of TARC was determined by ELISA and shown in box-plots. Horizontal lines represent the median; the box comprises the 25th and 75th percentiles and the error bars comprise the 10th and 90th percentiles. Outliers are indicated. Comparison among hr FDRs, lr FDRs, patients with RT1D-IRS, and healthy controls (ANOVA test).

Simultaneously, we found that the levels of TARC in hr FDRs were significantly higher, compared with lr FDRs and healthy controls, and similar to the levels detected in patients with R-T1D in IRS (438.83 ± 120.62 versus 312.04 ± 151.14 pg/mL, *P* < 0.05; 438.83 ± 120.62 versus 236.88 ± 89.19 pg/mL, *P* < 0.01; 438.83 ± 120.62 versus 398.14 ± 333.09 pg/mL, *P* = NS) (Figure 6). In addition, the level of TARC was significantly higher in patients with R-T1D in IRS than in healthy controls (*P* < 0.01) (Figure 6).

### 3.6. Binary Logistic Regression Analysis

This model has identified the levels of CXCR3^+^ and CCR4^+^ T memory cells and IP-10 as independent predictors for hr or lr in FDRs, in contrast to the TARC levels (Table 2).

## 4. Discussion

Our results have demonstrated that hr FDRs, defined by the presence of the autoantibodies, showed higher levels of CXCR3^+^ T cell subset and IP-10 chemokine, both associated with Th1 response, together with lower level of CCR4^+^ Th2 cell subset. In this study, complementary investigations imply that, in FDRs, the risk of progression to T1D might be strongly influenced by enhanced activity of Th1 and diminished activity of Th2 autoimmune response.

Until now, the studies examining the role of T cells subsets expressing different chemokine receptors in adult prediabetics were limited. In our study, the hr FDRs had higher number of T memory cells expressing Th1-associated chemokine receptor, suggesting the predomination of Th1 autoimmune response, which is in line with previous investigations in the FDRs of T1D patients [24, 25]. However, in another study in prediabetics, a reduction of Th1-associated chemokine receptor CXCR3 was demonstrated when both CD3^+^ and CD4^+^ T cells were analysed [21]. However, comparing that with our study, there are considerable differences in the subjects selection and methodology. The previous study has examined children only, while we included only adults and in that study all the subjects were evaluated within 12 months before the onset of T1D, in contrast to our subjects who were analysed earlier in prediabetic state, when Th1-mediated autoimmune response could be more intensive [3]. Moreover, from the methodological aspects, we analysed more specifically CD4^+^ T memory cells using four-color immunofluorescence staining, while the previous study was done in the total T cell populations with the use of three-color immunofluorescence staining, which might add to the difference between the results [21].

In addition, we demonstrated that the onset of T1D is characterized by the decreases in CXCR3^+^ Th1 memory T subsets, presumably reflecting possible recruitment of those cells in pancreatic tissue, in agreement with the findings reported previously [21, 26, 27].

In this context, the study done in adults with T1D in whole blood samples showed initial decrease in CXCR3/CCR4 expression ratio on CD4^+^ T cells [27]. Simultaneously, recently published study revealed interesting data about lower expression of Th1-associated chemokine receptors on circulated CD4^+^ but not on CD8^+^ T cells in T1D, suggesting suboptimal helper function at onset of the disease [26].

Having in mind that hyperglycemia and infection induce redistribution of lymphocytes subsets, in our study the blood samples were taken in state of euglycemia and we excluded patients with infections [28].

However, recent studies have revealed decreased expression of CXCR3 on CD4^+^ T cells in fulminant T1D onset, while increased CXCR3 expression levels were accompanied by mild T1D onset [29, 30], which is partly concordant with our findings, but awaits confirmation in the future studies of very close monitoring of the CXCR3 T cell changes at onset of T1D.

On the other hand, previous reports suggested higher CXCR3 expression on CD4loCD40^+^ T memory cells, unique T cell subpopulation, which is expanded in T1D [31], in contrast to our data. Still, the precise role of CD4lo T cells in T1D remains unclear, as some have observed CD4loCD40^+^ T cells to be highly diabetogenic [32], while others report that the CD4hi phenotype is a characteristic of the most diabetogenic T cells [33, 34]. However, we detected very low level of CD4lo subpopulation in lymphocytic gate as well as among CD4^+^ T cells. Having this in mind our further analysis was based on CD4hi, that is, CD4^+^ CD4^+^CD45RO^+^ T cells, in order not to overestimate the effect of CD4lo subpopulation on final results. Moreover, we did not determine CD40 marker in our study so we were not able to compare with the previous study using that marker. On the other hand, in contrast to that study, we included homogenous group of only R-T1D patients, in order to eliminate the effect of insulin therapy and disease duration on our results.

We found that hr FDRs had lower level of T memory cells expressing CCR4, Th2-associated receptor, compared to lr FDRs and healthy controls, and similar levels with patients with R-T1D in IRS.

Until now, to our knowledge, expression of CCR4^+^ chemokine receptor on T memory cells was not investigated in FDRs, but only in patients with R-T1D. In that context, it was previously demonstrated that CCR4^+^ on CD4^+^ T cells was decreased at the onset of T1D, which is in line with our data [21]. Recently, lower level of CCR4^+^ T cells and CCR4 mRNA was documented in infants with high genetic risk for T1D [35].

When we analyzed IP-10 level, hr FDRs showed the highest levels, similar to the level detected in patients with R-T1D, while patients with R-T1D had higher IP-10 level than healthy controls.

Recently, higher circulating levels of IP-10, IFN-*γ* and IL-18 were reported in patients with T1D, LADA, and FDRs positive for islet autoantibodies [27, 36–40], suggesting that IP-10 level might represent clinical marker of Th1 autoimmune response. These data implicate proinflammatory changes in cellular immunity in prediabetics which is in line with our observations. Simultaneously, this is in agreement with the data showing upregulation of two proinflammatory chemokines, CCL3 and CCL4, and downregulation of the functionally opposing chemokine CCL2 in high risk FDRs [39].

In contrast to these findings, other authors reported lack of difference in IP-10 levels or decreased IP-10 secretion in cell supernatants of patients with T1D compared to healthy controls [26], which might be the consequence of the selection of the patients at different stages of T1D showing differences in the amount of the residual beta cells.

Moreover, it was reported that IP-10 secreted from *β* cells activates and attracts autoreactive T cells and macrophages to the islets via CXCR3 receptor. These recruited cells produce numerous cytokines in the islets, further damaging *β* cells, and the accelerated IP-10 generation in residual *β*-cells is suggested to amplify *β*-cell destruction [41, 42]. Interestingly, recent experimental data suggest that inhibition of IP-10 could prevent T1D, while blockade of CXCR3 is not sufficient to stop T1D development, suggesting a more complex and still unclarified role of both IP-10 and CXCR3 T cell subset in the pathogenesis of the disease [43].

In our study, in contrast to our expectations, circulating TARC levels were higher, in hr FDRs than in lr FDRs and healthy controls, and similar to the level detected in patients with R-T1D. This finding signifies that TARC might represent an indirect marker of inflammation in pancreatic islets and that it might have a dual role with respect to its possible diabetogenicity, which was previously reported but remains yet unclarified [20].

In previous investigations, TARC levels were not analyzed in FDRs and rarely in T1D patients. It was reported that T1D patients had slightly higher levels than healthy controls [27].

The binary logistic regression analysis applied to our data has demonstrated that the levels of CXCR3^+^ and CCR4^+^ T memory cells, together with IP-10 chemokine levels, are independent predictors of the disease risk, high or low, in the nondiabetic FDRs.

Our results confirmed the dominant influence of Th1 autoimmune response in prediabetics, based on the increase in CXCR3^+^ and IP-10, together with diminished Th2 autoimmune response expressed as the decrease in CCR4^+^ T memory cells.

In conclusion, we have demonstrated the increases in CXCR3^+^ T memory cells in FDRs at high risk of developing T1D that may represent a potential target for the preventive intervention in the prediabetic state.

## Figures and Tables

**Table 1:** Characteristics of first degree relatives of patients with T1D (FDRs) at high risk (hr FDRs) and low risk (lr FDRs) for development of T1D, patients with recent-onset T1D (R-T1D) in insulin-requiring state (IRS), and healthy controls (HC) included in CD4^+^ T cells analysis in the peripheral blood.

Group	Number	Gender (m/f)	Age (years)	BMI (kg/m^2^)
hr FDRs	17	4/13	29.82 ± 8.83	23.71 ± 2.66
lr FDRs	34	18/16	26.44 ± 6.09	22.69 ± 3.72
RT1D-IRS	24	15/9	26.43 ± 6.02	21.40 ± 3.47
HC	18	2/16	28.18 ± 7.21	22.00 ± 4.21

**Table 2:** Independent factors related to first degree relatives (FDRs) at high risk (hr FDRs) or low risk (lr FDRs) for development of T1D in binary logistic regression analysis.

	Odds ratio (95% CI)	*P* value
IP-10	0.989–0.998	0.009
CXCR3^+^ CD4^+^ CD45RO^+^	0.431–0.833	0.002
CCR4^+^ CD4^+^ CD45RO^+^	1.229–2.319	0.001
TARC	0.002–0.005	0.342
